# Pinpointing disease genes through phenomic and genomic data fusion

**DOI:** 10.1186/1471-2164-16-S2-S3

**Published:** 2015-01-21

**Authors:** Rui Jiang, Mengmeng Wu, Lianshuo Li

**Affiliations:** 1 MOE Key Laboratory of Bioinformatics; Bioinformatics Division and Center for Synthetic & Systems Biology, TNLIST; Department of Automation, Tsinghua University, Beijing 100084, China; 2 Department of Statistics, Stanford University, CA 94305, USA

## Abstract

**Background:**

Pinpointing genes involved in inherited human diseases remains a great challenge in the post-genomics era. Although approaches have been proposed either based on the guilt-by-association principle or making use of disease phenotype similarities, the low coverage of both diseases and genes in existing methods has been preventing the scan of causative genes for a significant proportion of diseases at the whole-genome level.

**Results:**

To overcome this limitation, we proposed a rigorous statistical method called pgFusion to prioritize candidate genes by integrating one type of disease phenotype similarity derived from the Unified Medical Language System (UMLS) and seven types of gene functional similarities calculated from gene expression, gene ontology, pathway membership, protein sequence, protein domain, protein-protein interaction and regulation pattern, respectively. Our method covered a total of 7,719 diseases and 20,327 genes, achieving the highest coverage thus far for both diseases and genes. We performed leave-one-out cross-validation experiments to demonstrate the superior performance of our method and applied it to a real exome sequencing dataset of epileptic encephalopathies, showing the capability of this approach in finding causative genes for complex diseases. We further provided the standalone software and online services of pgFusion at http://bioinfo.au.tsinghua.edu.cn/jianglab/pgfusion.

**Conclusions:**

pgFusion not only provided an effective way for prioritizing candidate genes, but also demonstrated feasible solutions to two fundamental questions in the analysis of big genomic data: the comparability of heterogeneous data and the integration of multiple types of data. Applications of this method in exome or whole genome sequencing studies would accelerate the finding of causative genes for human diseases. Other research fields in genomics could also benefit from the incorporation of our data fusion methodology.

## Background

Pinpointing genes causative for inherited human diseases is the primary step towards the understanding of intrinsic mechanisms of such diseases. In the post-genomics era, the analysis of human genetic data is often combined with the mining of functional genomic data to facilitate the identification of potential causative genes [1,2]. For example, via genome-wide association (GWA) studies, genetic factors related to a query disease can typically be located within a region of 10M basepairs, containing about 100 candidate genes [3]. The problem is then how to rank these genes according to their strength of association with the query disease. Resorting to the whole-exome sequencing technique, dozens or hundreds of *de novo *mutations can be screened for a query disease [4]. The question is then how to infer true causative genes from candidate genes that contain such mutations.

Targeting on these demands, two groups of computational approaches have been proposed for the prioritization of candidate genes. The first group is designed based on the guilt-by-association principle, which suggests that genes associated with the same type of disease are similar in their functions [5]. Accordingly, candidate genes can be ranked according to their functional similarity to a set of seed genes that are known to be associated with the query disease. In existing studies belonging to this category, such similarities have been quantified based on gene expression [6], gene ontology [7], protein sequences [8], protein-protein interactions [9], and many others [10-12]. Methods have also been proposed to integrate multiple data sources for achieving high accuracy [13]. Nevertheless, the requirement of a predefined set of seed genes may greatly restrict the scope of applications of these methods, since according to the OMIM (Online Mendelian Inheritance in Man) database [14], genetic bases for a significant proportion of human diseases are completely unknown, making the selection of seed genes for such diseases a problem.

To overcome this limitation, the second group of methods, with the hallmark of using disease phenotype similarity data, has been proposed. For example, Lage et al. proposed a Bayesian model to integrate phenotypic similarities and protein-protein interaction (PPI) data [15]. Wu et al. suggested to quantify the strength of association between a disease and a gene using correlation between phenotype similarities and gene proximities [16]. Wu et al. further proposed to perform a local alignment of a phenotype network against a PPI network [17]. Li and Patra adopted a random walk with restart model on an integrated network composed of both diseases and genes [18]. Vanunu et al. proposed to simulate how disease status propagated through candidate genes [19]. Chen et al. proposed to quantify the strength of association between a disease and a gene using the maximum information flow in a phenome-interactome network [20]. These methods, though demonstrating higher accuracy and wider scope of applications than the guilt-by-association approaches, are often restricted by two factors: 1) the availability of the phenotype similarity data and 2) the coverage of the gene similarity data. For example, there are a total of 7,719 diseases recorded in the OMIM database till February 2014, whereas the most widely used phenotype similarity data as published in [21] covers only 5,080 (~66%) of such diseases. It is estimated that the human genome contains more than 20,000 genes, whereas the most widely used PPI data as published in [22] covers only 9,515 (< 50%) genes.

Motivated by these understandings, we propose a rigorous statistical model named pgFusion that integrates one type of phenotype similarity and seven types of gene similarities to pinpoint disease genes. The phenotype similarity data, which covers 7,719 diseases in the OMIM database, is derived using a text mining technique based on the Unified Medical Language System (UMLS) [23] and is the most comprehensive one among such data. The seven types of gene similarity data, including gene expression, gene ontology, pathway membership, protein sequence, protein domain, protein-protein interaction and regulation pattern, cover as many as 20,327 human genes, making the whole-genome scan of causative genes for a query disease possible. Based on these data, our method resorts to a linear regression model and a hypothesis testing procedure to derive 7 scores that quantify the strength of association between a query disease and a candidate gene from different perspectives, and further adopts the Fisher's method with dependence correction to combine these scores. We performed leave-one-out validation experiments to demonstrate the superior performance of pgFusion, and applied it to a real exome sequencing data set of epileptic encephalopathies [24], showing the capability of this approach in finding causative genes for complex disease. We finally provided the standalone software and user-friendly online services of our method at http://bioinfo.au.tsinghua.edu.cn/jianglab/pgfusion.

## Methods

### Workflow of pgFusion

**Figure 1:**
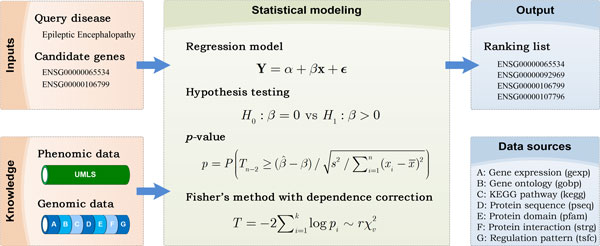
**Diagram of pgFusion**. Given a query disease and a list of candidate genes, pgFusion calculates the statistical significance that a candidate gene is causative for the query disease by integrating phenomic and genomic data, thereby providing a means of prioritizing the candidate genes.

The proposed method, named pgFusion, was designed based on the assumption that genes associated with diseases that shared common clinical traits would also share similar properties across multiple genomic data sources. As shown in Figure 1, inputs of this method included a query disease and a set of candidate genes, and the objective was to rank these genes according to their strength of association with the query disease. For this purpose, pgFusion relied on the OMIM and UMLS databases to calculate a phenotype similarity matrix for a total of 7,719 diseases and resorted to 7 genomic data sources (gene expression, gene ontology, KEGG pathway, protein sequence, protein domain, protein-protein interaction and regulation pattern) to derive 7 gene functional similarity matrices for a total of 20,327 human genes. With such phenomic and genomic information available, pgFusion resorted to a regression model and the Fisher's method to examine one candidate gene at a time. In the regression model, pgFusion explained the phenotype similarity between two diseases using their genotype similarity, which was defined as the total functional similarities of their associated genes under a certain genomic data source. The strength of association between the query disease and a candidate gene was then assessed by a hypothesis testing procedure and quantified by the corresponding *p*-value. Final results were then 7 *p*-values, one for a genomic data source. In the Fisher's method, pgFusion integrated the 7 *p*-values to calculate a single *p*-value, with the consideration of the dependence between these *p*-values. A multiple testing correction procedure was then applied to the final *p*-values of all candidate genes to control the positive false discovery rate of the results by calculating *q*-values from *p*-values. Finally, candidate genes were sorted according to their *q*-values to produce the output ranking list.

### Derivation of phenotype similarity

We adopted the text mining technique to derive pairwise phenotype similarity between diseases. Briefly, we first extracted a total of 7,719 disease records from the OMIM database and split sentences in the TX and the CS fields of these records into words. Then, we mapped these words onto concepts in the UMLS database by using the MetaMap program [25]. Next, for each OMIM record, we counted the frequency of occurrence of each concept in the record, obtaining a high dimensional numeric vector. Finally, we calculated pairwise phenotype similarity between diseases as the cosine of the angle between corresponding vectors. We assessed relationships between the phenotype similarity derived this way and several genotype similarities, and we found strong evidence to support the existence of correlations between the phenotype and genotype similarities.

### Derivation of gene similarities

We derived gene functional similarity scores from 7 types of genomic data, including gene expression, gene ontology, pathway membership, protein sequence, protein domain, protein-protein interaction and regulation pattern. Each of such scores ranged from 0 to 1, denoting the lowest and highest similarities, respectively.

#### Gene expression

Focusing on whole-genome microarrays for a total of 44,775 transcripts across 79 tissues [26], we characterized each human gene with a 79-dimensional numeric vector that represented expression levels of the gene across the tissues. For a pair of two genes, we calculated the absolute value of the Pearson's correlation coefficient of the corresponding vectors to obtain their raw similarity scores. Considering that such raw scores may include noise in the original expression data, we further applied an exponential transformation to convert raw scores into final similarity scores, as

$$\varphi_{gh}^{\left(gexp\right)}=\text{exp}\left[-{\left(\frac{1-\omega_{gh}^{\left(gexp\right)}}{\sigma^{\left(gexp\right)}}\right)}^{2}\right],$$

where $\varphi_{gh}^{\left(gexp\right)}$ was the final score for two genes *g *and *h*, $\omega_{gh}^{\left(gexp\right)}$ the raw score, and *σ*^(gexp) ^the standard deviation of raw scores for all gene pairs. With this transformation, the highest raw score (1.0) kept highest, while the lowest raw score (0.0) became exp[-(*σ*^(gexp)^)^-2^], which was close to zero because the standard deviation *σ*^(gexp) ^was typically small.

#### Gene ontology

Focusing on the biological process domain of the gene ontology and associated annotations [27], we collected a total of 25,616 concepts in the annotations and characterized each human gene using a numeric vector of such number of dimensions, with each element being the information content of the corresponding concept. For a pair of two genes, we calculated the cosine of the angle between the corresponding vectors to obtain their raw similarity scores and further applied the aforementioned exponential transformation to convert raw scores into final similarity scores. Note that although there have been quite a few methods for calculating gene semantic similarity based on the gene ontology [28], it has been shown recently that the cosine measure, though simple, often produces reasonable results [29].

#### Pathway membership

Focusing on human pathways in the KEGG database [30] and discarding diseases-related ones to avoid biases towards well-studied diseases, we obtained a total of 238 pathways and characterized each human gene using a binary vector of such number of dimensions. For a pair of two genes, we calculated the cosine of the angle between the corresponding vectors to obtain their raw similarity scores and further applied the exponential transformation to obtain final similarity scores.

#### Protein sequence

We extracted a total of 20,274 human protein sequences from the Swiss-prot database [31] and ran the Smith-Waterman algorithm implemented in SSEARCH [32] to obtain their pairwise local sequence alignments. Then, we constructed a sequence similarity network of these proteins by connecting two proteins with an undirected edge if their alignment e-value is less than a predefined threshold (10^-4^). Next, we calculated the shortest path distance ($\delta_{gh}^{\left(pseq\right)}$) for every pair of proteins (*g *and *h*) in this network and converted it to a similarity value in the range of 0 and 1 ($\omega_{gh}^{\left(pseq\right)}=1-\delta_{gh}^{\left(pseq\right)}/\text{max}\delta_{gh}^{\left(pseq\right)}$). Finally, we applied the exponential transformation to obtain the similarity score. Note that the construction of a sequence similarity network in this procedure greatly reduced the sensitivity to the parameters involved and thus enhanced the robustness of this method.

#### Protein domain

We obtained a total of 14,831 domains from the Pfam database (Version 27.0) [33] and characterized each human protein using a binary vector of such number of dimensions. For a pair of two genes, we calculated the cosine of the angle between the corresponding vectors to obtain their raw similarity scores and further applied the exponential transformation to obtain final similarity scores.

#### Protein-protein interaction

We extracted a total of 403,514 interactions among 13,747 proteins from the STRING database (Version 9.1) [34] and constructed a protein-protein interaction network accordingly. Then, we calculated the shortest path distance ($\delta_{gh}^{\left(strg\right)}$) for every pair of proteins (*g *and *h*) in this network and converted it into a value in the range of 0 and 1 ($\omega_{gh}^{\left(strg\right)}=1-\delta_{gh}^{\left(strg\right)}/\text{max}\delta_{gh}^{\left(strg\right)}$). Finally, we applied the exponential transformation to obtain the similarity score.

#### Regulation pattern

We extracted a total of 218 high confidence position specific scoring matrices for the same number of vertebrate transcription factors from the TRANSFAC database [35]. We then searched 1,000 basepairs upstream for each human gene using the program MATCH to identify potential binding sites for each transcription factor. Next, we characterized each gene using a numeric vector of 218 dimensions, with each element indexing the number of potential binding sites for the corresponding transcription factor. Finally, for each pair of two genes, we calculated the cosine of the angle between the corresponding vectors to obtain their raw similarity scores and further applied the exponential transformation to obtain final similarity scores.

### Scoring association strength by regression and hypothesis testing

Given the phenotype similarity matrix and a gene functional similarity matrix derived from a type of genomic data, we adopted a linear model as proposed in the literature [16] to explain the phenotype similarity between two diseases using functional similarities of genes associated with the diseases, as

$$Y_{de}=\alpha +\beta x_{de}+\varepsilon_{de},$$

where *d *and *e *indexes two diseases, *Y_de _*their phenotype similarity, *ε_de _*Gaussian noise, and *x_de _*their genotype similarity defined as

$$x_{de}=\underset{g\in D}{\sum }\underset{h\in E}{\sum }\varphi_{gh},$$

with **D **and **E **being sets of genes known as associated with diseases *d *and *e*, respectively, and *φ_gh _*the functional similarity between genes *g *and *h *according to the genomic data in use.

Particularly, suppose *d *to be the query disease and *g *a candidate gene, we assumed *g *would be the only gene associated with *d *and wrote a regression model as

Y=α+βx+ε,

where *α *and *β *are regression intercept and slope, respectively, $Y={{\left(Y_{d1},\ldots ,Y_{dn}\right)}^{T}}_{n\times 1}$ the vector composed of phenotype similarities between *d *and all other *n *diseases in the similarity matrix, $x={{\left(x_{d1},\ldots ,x_{dn}\right)}^{T}}_{n\times 1}$ the vector of corresponding genotype similarities with $x_{di}=\sum_{k\in I_{i}}\varphi_{gk}$ and **I***_i _*the set of genes known as associated with the *i*-th disease for *i *= 1,...,*n*, and *ε *= (*ε*_1_,...,*ε_n_*)*^T ^*with *ε_i _*~ *N*(0,*σ*^2^) independent and identically distributed for *i *= 1,...,*n*.

With this regression model, we quantified the strength of association between the query disease *d *and the candidate gene *g *using the statistical significance of the hypothesis testing problem

$$H_{0}:\beta =0\text{ vs }H_{1}:\beta >0.$$

Define the test statistic *T *as,

$$T=\frac{\hat{\beta }}{\sqrt{S^{2}/\sum_{i=1}^{n}{\left(x_{i}-\bar{x}\right)}^{2}}},$$

where $S^{2}=\sum_{i=1}^{n}{\left(Y_{i}-\hat{\alpha }-\hat{\beta }x_{i}\right)}^{2}/\left(n-2\right)$, $\hat{\beta }=\sum_{i=1}^{n}\left(x_{i}-\bar{x}\right)\left(Y_{i}-Ȳ\right)/\sum_{i=1}^{n}{\left(x_{i}-\bar{x}\right)}^{2}$ and $\hat{\alpha }=Ȳ-\bar{x}\hat{\beta }$. It is obvious that the statistic has a student's *t *distribution with *n*-2 degrees of freedom under the null hypothesis and the normal assumption. The *p*-value of the proposed test can then be calculated as *P*(*T*_*n*-2 _≥ *t*) with *t *the realized value of the statistic.

However, in the case that the normal assumption does not hold, the *p*-value obtained from the *t *distribution may not reliably reflect the true statistical significance. We therefore calibrated the *p*-value by simulating the distribution of raw *p*-values for all disease-gene pairs that were not included in annotated associations and calculating the adjusted *p*-value as the proportion of raw *p*-values in this distribution that was smaller than or equal to the raw *p*-value need to be calibrated.

### Fusion of association scores for multiple genomic data sources

We adopted Fisher's method to integrate *p*-value derived from different types of genomic data to obtain a single score, with an extra effort on the correction of dependence between the *p*-values.

Specifically, given the *p*-values to be combined, denoted by *p*_1_,...,*p_k_*, where *k *= 7 is the total number of data sources, we defined the fisher's statistic as

$$X=\overset{k}{\underset{i=1}{\sum }}V_{i}\;\text{with}\;V_{i}=-2\text{log}p_{i}.$$

It is clear that under the null hypothesis, *p_i _*~ Uniform(0,1) and $V_{i}\sim \chi_{2}^{2}$. In the independent case, it is obvious that $\sum_{i=1}^{k}V_{i}\sim \chi_{2k}^{2}$. In the dependent case, we follow the literature [36] to assume that *T *follows a scaled chi-squared distribution as $X=\sum_{i=1}^{k}V_{i}\sim r\chi_{v}^{2}$. The problem is therefore how to estimate the scale *r *and the degrees of freedom *v*. Resorting to the method of moments, population mean and variance are given as

$$\text{E}[r\chi_{v}^{2}]=rv\text{ and Var}[r\chi_{v}^{2}]=2r^{2}v,$$

while the corresponding sample mean and variance are derived as

$$\text{E}[X]=2k\text{ and Var}[X]=\sum_{i=1}^{k}\sum_{j=1}^{k}\mathrm{cov}(V_{i},V_{j}).$$

Matching these quantities for the population and the sample, we obtain

$$\hat{r}=\frac{1}{4k}\overset{k}{\underset{i=1}{\sum }}\overset{k}{\underset{j=1}{\sum }}\text{cov}\;\left(V_{i},V_{j}\right)\;\text{and}\;\hat{v}=2k/\hat{r}.$$

Covariances cov(*V_i_*,*V_j_*) can be estimated using a normal model as follows. Suppose *p_i _*= Φ(1 - *z_i_*), where Φ(·)is the cumulative distribution function of the standard normal distribution and *Z_i _*a statistic that has a standard normal distribution under the null hypothesis. As suggested in the literature [36], let

$${\hat{\rho }}_{ij}=\text{Cor(}Z_{i},Z_{j})\text{ and }{\tilde{\rho }}_{ij}={\hat{\rho }}_{ij}\left(1+\frac{1-{\hat{\rho }}^{2}{}_{ij}}{2n-1}\right).$$

The covariance is then calculated as

$$\text{Cov}(V_{i},V_{j})\approx a_{1}{\tilde{\rho }}_{ij}+a_{2}{\tilde{\rho }}^{2}{}_{ij}+a_{3}{\tilde{\rho }}^{3}{}_{ij}+a_{4}{\tilde{\rho }}^{4}{}_{ij},$$

where $a_{1}=3.263119,a_{2}=0.709866,a_{3}=0.026589,a_{4}=-0.709866/n$, *n *the sample size for obtaining *Z_i_*.

We further applied multiple testing corrections to the combined *p*-values by controlling the positive false discovery rate (pFDR) of candidate genes through their *q*-values [37]. Existing studies have shown the significant improvement in the test power of this method over the traditional approach of Benjamini-Hochberg that controls the false discovery rate (FDR) [38]. It is possible that some data sources are absent for a candidate gene. To deal with this problem, we ignored the missing data source in the Fisher's method and decreased the total number of *p*-values to be combined accordingly.

## Results

### Data sources

We extracted a total of 7,719 diseases from the OMIM database (accessed in February 2014) and derived pairwise phenotype similarities of these diseases by applying the text mining technique to their OMIM records with the use of UMLS (version 2014AA) as the standard vocabulary. We extracted a total of 4,368 associations between 3,709 of these diseases and 2,870 genes using the tool BioMart [39].

We obtained gene expression data that measured whole genome transcripts across 79 human tissues from the literature [26] and derived pairwise expression similarities (gexp for short) between 12,462 genes. We extracted the biological process domain of the gene ontology and associated annotations for human genes (both released on 2014-02-13), and we derived pairwise semantic similarities (gobp) between 14,465 genes. We downloaded a total of 283 KEGG pathway for human (released on 2014-03-11) and derived pairwise pathway similarities (kegg) between 6,468 genes. We extracted a total of 20,272 human protein sequences from the Swiss-prot database (release 2014_01) and derived pairwise sequence similarities (pseq) between 14,196 genes. We extracted a total of 14,831 protein domains from the Pfam database (version 27.0) and derived pairwise domain similarities (pfam) between 17,091 genes. We extracted a total of 403,514 interactions between 13,747 human proteins from the STRING database (version 9.1) and derived pairwise network similarities (strg) between 12,432 genes. We extracted high quality position specific scoring matrices for 218 vertebrate transcription factors from the TRANSFAC database (release 2013.1) and derived pairwise regulation similarities (tsfc) between 20,314 genes. Putting together, we obtained a total of 20,327 genes that were present in at least one of the 7 data sources. The method for deriving each type of gene similarity is detailed in the method section. The coverage of each data source is shown in Table 1.

**Table 1:** Coverage and accuracy of individual data sources.

Data source	Coverage		Linkage Interval			Random Control		
			
	Genes	Ratio (%)	TOP (%)	MRR (%)	AUC (%)	TOP (%)	MRR (%)	AUC (%)
**gexp**	**12,462**	**61.31**	**49.43**	**23.99**	**76.47**	**50.32**	**23.62**	**76.87**
gobp	14,465	71.16	77.47	11.94	88.56	78.48	11.18	89.35
kegg	6,468	31.82	52.98	15.27	85.85	53.73	14.20	86.97
pseq	14,196	69.84	51.21	18.55	82.12	51.85	17.76	82.96
pfam	17,091	84.08	60.87	15.86	84.83	62.25	14.52	86.25
strg	12,432	61.16	72.99	12.64	88.04	73.60	11.84	88.90
tsfc	20,314	99.94	52.13	26.38	74.97	51.90	26.19	74.48
**all**	**20,327**	**100.0**	**79.65**	**9.45**	**91.37**	**81.85**	**9.94**	**90.48**

Results are obtained according to the leave-one-out cross-validation experiments.

### Phenotype similarity correlates with genotype similarity

**Figure 2:**
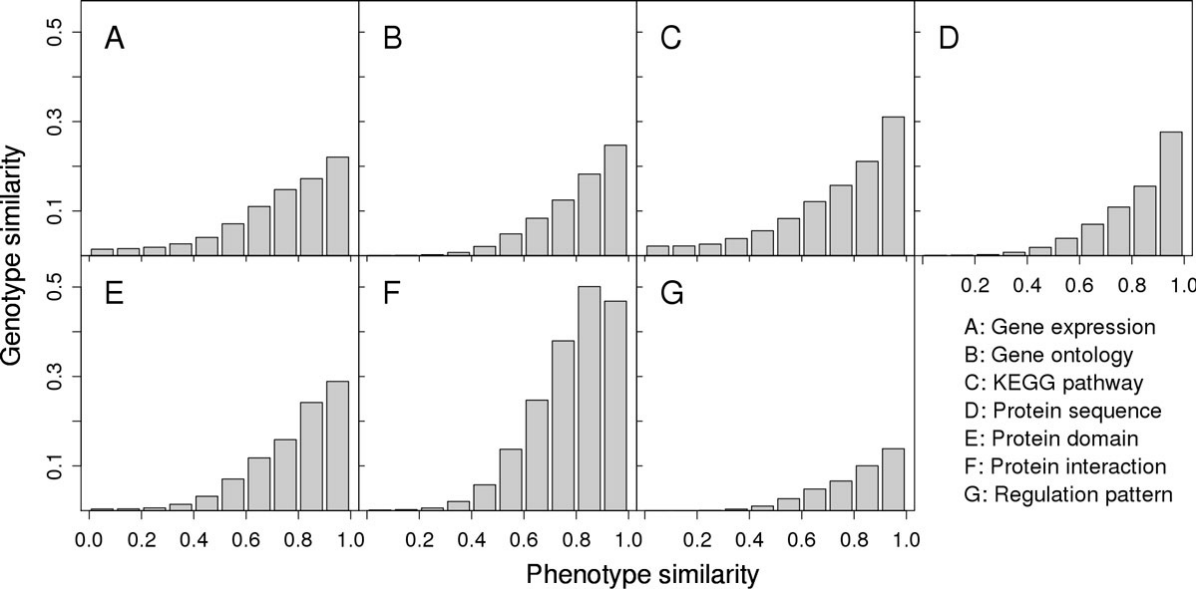
**Phenotype similarity correlates with genotype similarity**. For a pair of two diseases, we define their phenotype similarity as the cosine value calculated by the text mining technique and their genotype similarity under a certain genomic data source as the total pairwise similarity of their associated genes derived from the genomic data.

We first validated whether the derived phenotype similarity was correlated with genotype similarities according to annotated associations between diseases and genes. For a pair of two diseases, we defined their phenotype similarity as the cosine value calculated by the text mining technique and their genotype similarity under a certain genomic data source as the total pairwise similarity of their associated genes derived from the genomic data. With these definitions, we calculated the phenotype similarity between each pair of the 3,709 diseases with associated genes, partitioned the resulting 6,876,486 values into 10 equal bins, averaged over genotype similarities of disease pairs in each bin, and plotted the resulting relationships between phenotype and genotype similarities in Figure 2.

From the figure, we clearly see strong correlation between phenotype similarity and genotype similarity derived from each of the 7 genomic data sources. Taking gene expression as an example (Figure 2A), for disease pairs with very weak phenotype similarity (0.0~0.1), the genotype similarity is only 0.0145 on average. For disease pairs with strong phenotype similarity (0.9~1.0), the genotype similarity is as high as 0.2204 on average. For disease pairs with medium phenotype similarity (0.4~0.5), the genotype similarity is also at the medium level (0.0409). Furthermore, it is obvious that with the increase of the phenotype similarity, the genotype similarity also increases. For the other 6 genomic data, we observe similar pattern. These results suggest that diseases having weak phenotypic overlap tend to have small genotypic overlap, while diseases having strong phenotypic overlap tend to have large genotypic overlap, in accord with one of our previous analysis [17].

To quantitatively measure the correlation between phenotype similarity and genotype similarity, we derived for each genomic data source two vectors, one composed of mean phenotype similarities of disease pairs in the 10 bins and the other consisting of corresponding mean genotype similarities. We then calculated Pearson's correlation coefficient of these two vectors for each type of data. Results show that the correlation coefficients are 0.9626 (*p*-value = 8.193 × 10^-6^) for gene expression, 0.9341 (*p*-value = 7.607 × 10^-5^) for gene ontology, 0.9404 (*p*-value = 5.133 × 10^-5^) for KEGG pathway, 0.8987 (*p*-value = 4.076 × 10^-4^) for protein sequence, 0.9449 (*p*-value = 3.778 × 10^-5^) for protein domain, 0.9408 (*p*-value = 4.994 × 10^-5^) for protein-protein interaction, and 0.9322 (*p*-value = 8.512 × 10^-5^) for regulation pattern. We then conclude that the phenotype similarity positively correlates with the genotype similarity with strong statistical significance.

### Data fusion improves prioritization performance

We then validated pgFusion using the 4,368 annotated associations between 3,709 diseases and 2,870 genes by a large-scale leave-one-out cross-validation experiment against a linkage interval. In each validation run, we focused on one disease-gene pair in an annotated association and saw whether our method can correctly identify the gene from a set of control genes. For this purpose, we took the disease as the query disease and the gene as the test gene, collected a set of 99 control genes that had the shortest distance to the test gene among all genes in the same chromosome as the test one, and ranked the test gene against the control genes using our method. In this procedure, we removed all annotated associations between the query disease and genes in the regression model to simulate the circumstance that the genetic basis of the query disease is completely unknown.

**Figure 3:**
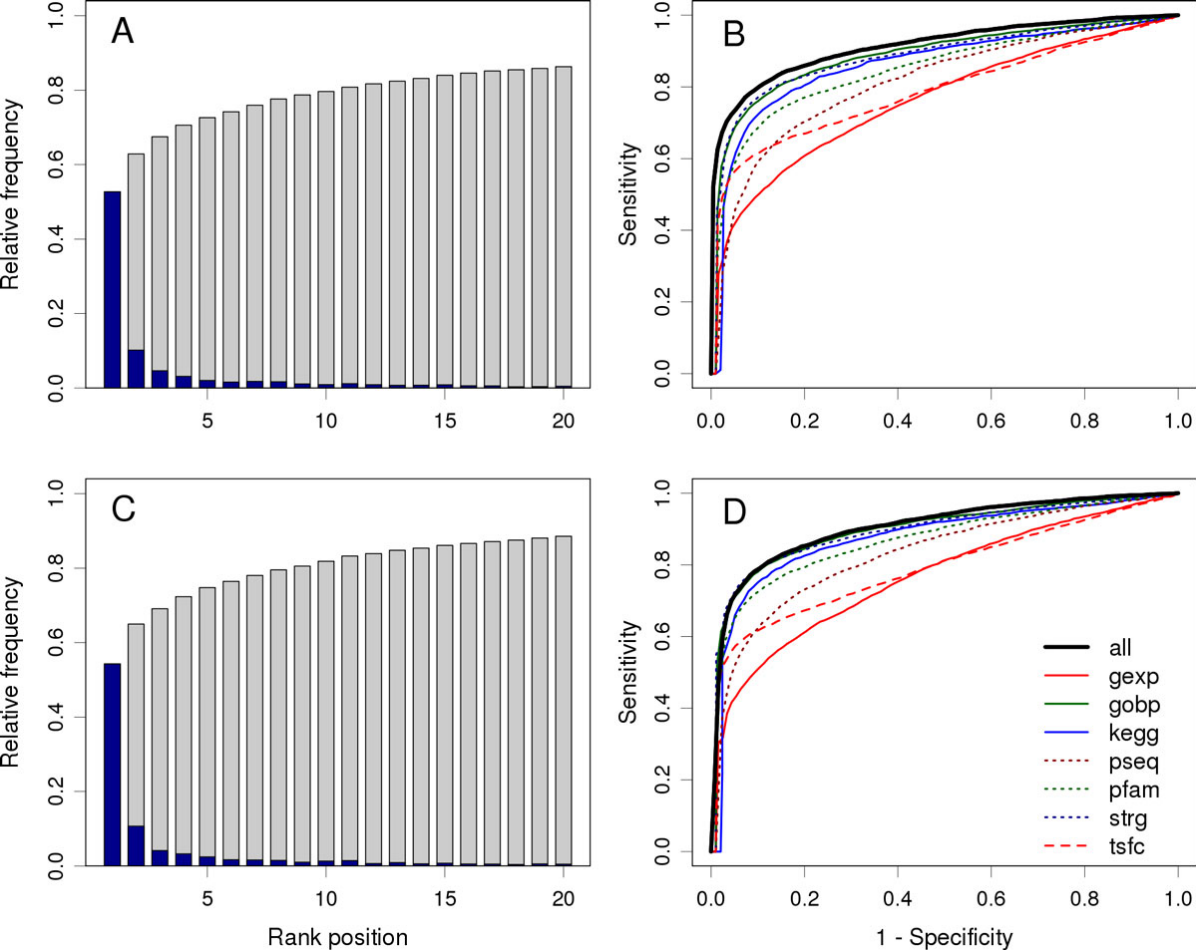
**Performance of pgFusion**. (A) and (C) Probability mass (dark blue) and cumulative distribution (gray) of genes ranked among top 20 for validation against a linkage interval and random controls, respectively. (B) and (D) ROC curves for validation against a linkage interval and random controls, respectively.

We summarized ranks of the test genes in Figure 3(A). In a total of 4,368 validation runs, pgFusion ranked 2,295 test genes at the top and 3,479 among top 10. In contrast, with a random guess procedure, one can only expect 4,368/100≈43.7 test genes ranked at the top and 10 × 4,368/100≈436.8 enriched among top 10. These results suggest the capability of our method in identifying disease genes from a linkage interval. We then derived two criteria to quantify the performance of pgFusion. Dividing the rank of a test gene by the total number of test and control genes in a validation run, we obtained the rank ratio of the test gene. Averaging rank ratios of all test genes, we obtained the first criterion called the Mean Rank Ratio (MRR). At a certain threshold of the rank ratio, we defined the sensitivity and the specificity as the fraction of test and control genes ranked above and below the threshold, respectively. Varying the threshold, we plotted the rank operating characteristic (ROC) curve (sensitivity versus 1-specificity) and further calculated the area under this curve as the second criterion called the AUC score. As shown in Figure 3(B), the ROC curve of pgFusion (black solid line) climbs fast towards the upper left corner of the plot, suggesting the capability of this method in achieving high sensitivity while maintaining high specificity. As shown in Table 1, the MRR and AUC for the 4,368 validation runs are 9.45% and 91.37% respectively. These results further suggest the effectiveness of our method, considering that random guess can only yield an MRR of 50% and an AUC of 50%.

We then compared the performance of pgFusion with that of individual genomic data sources. As shown in Table 1, among the 7 data sources, the gene ontology (gobp) yields the highest performance (MRR = 11.94%, AUC = 88.56%), followed by the protein-protein interaction (strg) (MRR = 12.64%, AUC = 88.04%). The regulation pattern (tsfc) yields the lowest performance (MRR = 26.38%, AUC = 74.97%), followed by the gene expression (gexp) (MRR = 23.99%, AUC = 76.47%). The improvements of pgFusion over individual data sources are then as high as 64.19% when compared with the regulation pattern and as low as 20.89% when compared with the gene ontology, in terms of the MRR. These results clearly demonstrate the vast improvement of pgFusion over individual genomic data sources in the prioritization accuracy and suggest the power of data fusion.

In exome sequencing studies, genetic variants are sequence across the whole exome, it is therefore necessary to validate whether pgFusion is capable of identifying disease genes for a query disease from candidate genes spreading over the entire genome. For this purpose, we performed a large-scale leave-one-out cross-validation experiment against random controls. Specifically, in each validation run, we focused on one disease-gene pair in an annotated association, took the disease as the query disease and the gene as the test gene, collected a set of 99 control genes that were selected at random from the entire genome, and ranked the test gene against the control genes using our method. We also removed all annotated associations between the query disease and genes in the regression model to pretend that the genetic basis of the query disease is completely unknown. We summarized ranks of the test genes in this validation in Figure 2(C). In a total of 4,368 validation runs, pgFusion ranked 2,371 test genes at the top and 3,575 among top 10. Considering that a random guess procedure can only rank 43.7 test genes ranked at the top and 436.8 genes among top 10, the capability of our method in identifying disease genes from random controls is strongly supported. Besides, the low MRR (9.94%) and high AUC (90.48%) as shown in Table 1, together with the fast climbing shape of the ROC curve in Figure 3(D), further confirm the effectiveness of our method in this validation. Furthermore, comparison with individual data sources, as shown in Table 1, also demonstrate the vast improvement in the performance of pgFusion. For example, the improvements of pgFusion over the gene ontology (gobp) is 11.19% in terms of the MRR.

More importantly, the coverage of pgFusion also benefits from data fusion. For example, as shown in Table 1, KEGG covers only 6,468 genes. Gene ontology (gobp) covers 14,465 genes. Protein-protein interaction (strg) covers 12,432 genes. Regulation pattern (tsfc), though covers 20,314 genes, can only achieve the lowest accuracy. With data fusion, however, pgFusion covers 20,327 genes, much more than most individual data sources, and thus makes it feasible to perform a whole-genome scan for disease genes for a query disease.

### Contributions of individual data sources

**Figure 4:**
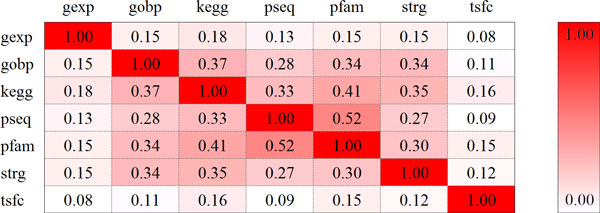
**Pearson's correlation coefficients between individual data sources**.

We presented pairwise Pearson's correlation coefficient of *p*-values produced by the 7 genomic data sources in Figure 4. Briefly, gene expression (gexp) and regulation pattern (tsfc) exhibit weak correlations with the other 5 data sources, which however show medium pairwise correlations. This evidence suggests the necessity of performing dependence correction in the Fisher's combine probability test.

**Figure 5:**
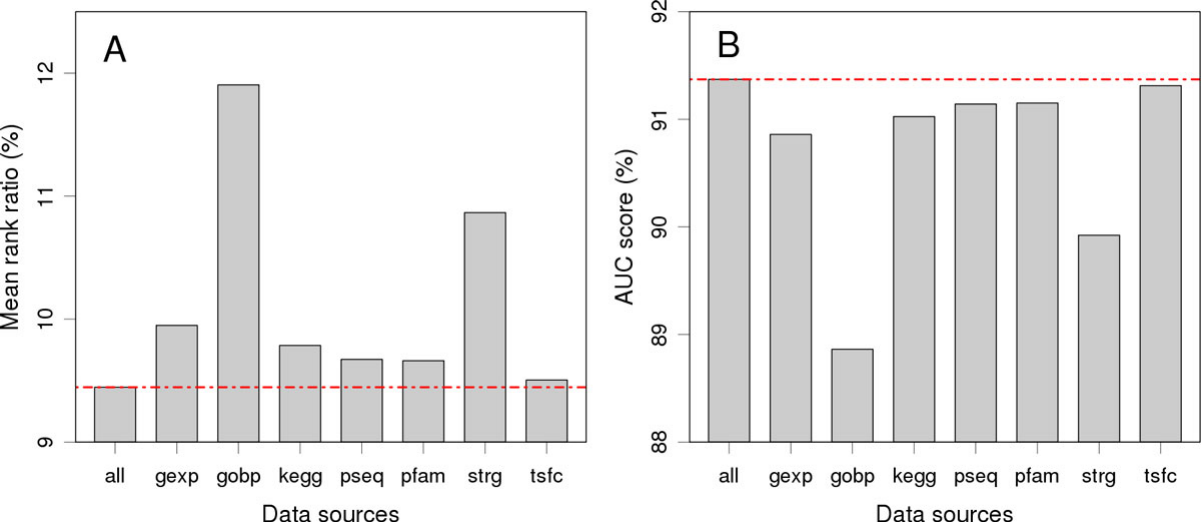
**Contributions of individual data sources**. Results are obtained by excluding individual data sources in the calculation of the combined *p*-value.

Considering the existence of such correlations, the prediction power of an individual data source may not reflect its real contribution to the final performance of our method. We therefore evaluated relative contribution of a data source by erasing the data source from the Fisher's method and repeating the validation experiment against a linkage interval. As shown in Figure 5, for each of the 7 genomic data sources, the MRR increases while the AUC decreases after the removal of the data source, suggesting its positive contribution. In detail, the gene ontology exhibits the highest contribution because with its removal the MRR increases from 9.45% to 11.90%. The protein-protein interaction exhibits the second highest contribution since its removal resulted in an increment of MRR from 9.45% to 10.87%. It is also interesting to see that the removal of the gene expression resulted in an increment of MRR from 9.45% to 9.95%, suggesting this data sources has the third highest contribution. However, using this data source alone only yields the second worst performance (Table 1). We conjecture this inconsistency is due to the fact that the gene expression exhibits weak correlations between the other data sources, and thus information provided by this data source could complement that provided by the others to facilitate the accurate prioritization of candidate genes.

### Comparison with existing methods

We compared the performance of pgFusion with that of two representative methods for gene prioritization, Cipher [16] and Endeavour [13]. Briefly, Cipher represents a category of methods that rely on a single source of phenomic data and a single source of genomic data. This method is mathematically equivalent to our approach when using PPI information only. Therefore, it is obvious that our method outperforms Cipher in all evaluation criteria, as demonstrated in Table 1 and analysed in the above section.

Endeavour represents another category of methods that rely on multiple sources of genomic data to prioritize genes. This method was developed according to the guilt-by-association principle [5] and thus required a set of seed genes known to be associated with a query disease as an extra input [13]. To meet this requirement, for a query disease, we resorted to the phenotype similarity data to select 5 to 20 diseases that owned the highest phenotype similarities with the query disease and then used genes known as associated with these diseases as seed genes for the query disease. We repeated the leave-one-out cross-validation experiment against a linkage interval for Endeavour, using the same 7 sources of genomic data. Results show that Endeavour achieves the highest performance (MRR = 16.61% and AUC = 83.64%) when seed genes are obtained from 20 diseases that are most similar to the query one. When 5, 10 and 15 most similar diseases are used to obtain seed genes, Endeavour achieves MRRs of 18.40%, 18.17% and 17.11%, respectively and AUCs of 81.86%, 82.09% and 83.14%, respectively. All these criteria are much lower than those achieved by pgFusion (MRR = 9.45% and AUC = 91.37%). We conjecture this observation can probably be attributed to the fact that pgFusion uses phenomic data in a global way, while in our experiment Endeavour only partially uses such information.

### Application to exome sequencing studies

Recent advancements in exome sequencing studies have demonstrated that the collection of *de novo *mutations affecting different genes in different individuals might explain a proportion of such common complex diseases as epileptic encephalopathies [24]. We therefore apply our method to the exome sequencing data of this complex disease to demonstrate the power of our method in diagnosing disease genes.

Epileptic encephalopathies (MIM: 615369) refer to a group of severe childhood epilepsy disorders for which the cause remains largely unknown [24]. These disorders typically affect the cognitive and behaviour of the patients, especially infant and children, and sometimes may cause an early death. Recently, exome sequencing was successfully applied to the study of this group of complex diseases, showing strong statistical evidence on the association of several *de novo *mutations with epileptic encephalopathies (PMID 23934111) [24]. From the sequencing data of 264 probands and their parents in this study, we collected 179 unique candidate genes that contained 192 unique *de novo *mutations, and 19 of these genes were reported as likely functional in the literature [24]. When looking at the results produced by our method with the assumption that genetic bases of this disease is completely unknown (Table 2), we observe that all genes ranked among top 5 have been reported as likely functional, yielding a *p*-value of 8.03 × 10^-6 ^according to the one-sided Fisher's exact test against the alternative hypothesis that the probability of observing 5 functional genes among top 5 is significantly higher than the random guess. Moreover, 9 genes ranked among top 10 and 14 genes ranked among top 20 are likely functional, yielding *p*-values of 2.06 × 10^-9 ^and 1.60 × 10^-12^, respectively. At the *q*-value cut-off value 0.001, 7 out of 8 candidate genes are likely functional, yielding a *p*-value of 3.65 × 10^-7^. At the *q*-value cut-off value 0.01, 10 out of 11 candidate genes are likely functional, yielding a *p*-value of 1.34 × 10^-10^. All these results strongly support the capability of our method in identifying disease genes for this complex disease.

**Table 2:** Top 20 candidate genes for epileptic encephalopathies.

Rank	Chromosome	Gene	*p*-value	*q*-value	Functional
**1**	**2**	**SCN2A**	**1.03E-06**	**0.000118**	**Yes**
**2**	**5**	**GABRA1**	**1.38E-06**	**0.000118**	**Yes**
**3**	**2**	**SCN1A**	**2.04E-06**	**0.000118**	**Yes**
**4**	**15**	**GABRB3**	**6.37E-06**	**0.000277**	**Yes**
**5**	**9**	**KCNT1**	**1.03E-05**	**0.000359**	**Yes**
**6**	**20**	**KCNQ2**	**1.46E-05**	**0.000423**	**Yes**
7	4	GABRB1	3.07E-05	0.000702	−
**8**	**8**	**KCNQ3**	**3.23E-05**	**0.000702**	**Yes**
**9**	**12**	**SCN8A**	**6.59E-05**	**0.001153**	**Yes**
**10**	**9**	**STXBP1**	**6.62E-05**	**0.001153**	**Yes**
**11**	**X**	**ALG13**	**0.000256**	**0.004053**	**Yes**
**12**	**X**	**CDKL5**	**0.005170**	**0.074969**	**Yes**
13	20	KCNB1	0.008351	0.111775	−
**14**	**10**	**ANK3**	**0.010374**	**0.128932**	**Yes**
15	11	FAM86C1	0.012323	0.138576	−
16	5	GPR98	0.013428	0.138576	−
**17**	**9**	**GRIN1**	**0.013539**	**0.138576**	**Yes**
18	1	NFASC	0.015651	0.151292	−
19	12	CUX2	0.016939	0.155127	−
**20**	**12**	**GRIN2B**	**0.018004**	**0.156637**	**Yes**

Genes marked as functional are those that have been reported as likely functional in the literature (PMID 23934111).

### Whole-genome scan of disease genes

We further performed a whole-genome scan of causative genes for a total of 7,719 diseases in the phenotype similarity matrix. Focusing on genes collected in either of the seven genomic data sources, we extracted a total of 20,327 genes that spread over the entire genome and applied pgFusion to score these genes for each disease. Prediction results, together with an online service and the standalone software of pgFusion, are available at http://bioinfo.au.tsinghua.edu.cn/jianglab/pgfusion.

## Conclusions and discussion

In this paper, we have proposed a bioinformatics approach called pgFusion that integrated one type of phenotype similarity and seven types of gene similarities for the inference of disease genes. The success of our method can be attributed to the carefully designed statistical model that relates the calculation of association strength to a hypothesis testing problem and combines multiple data sources with the consideration of their pairwise correlations. Grounded on the theoretical modelling, our method achieves not only high coverage but also superior accuracy, thereby providing a practical way in such analysis as the prioritization of candidate genes in whole-exome sequencing studies.

Certainly, our method can further be improved from the following aspects. First, although we currently focus on UMLS to derive phenotype similarity, other standard vocabularies such as the Medical Subject Headings (MeSH) and the human phenotype ontology (HPO) can also be adopted. Second, most existing methods for prioritizing candidate genes so far do not explicitly address the possible bias towards well-studied genes. This bias issue is alleviated with the integration of multiple types of data, because ddifferent data sources measure gene functions from different points of view and do not depend on a single type of data to make inference. However, how to explicitly eliminate the influence of bias is still an open question worth exploration. Third, we currently do not weight different data sources. Although theoretically it is not hard to assign different weights to different data sources in Fisher's method, how to determine these weights is itself a problem that needs careful exploration. Finally, in the era of big data, the integration of multiple types of heterogeneous data is itself an important problem, the method we used in this paper provides a means for solving two basic questions, the comparability of heterogeneous data and the integration of multiple types of data. How to incorporate our method into other research fields in systems biology is one of our future focuses.

## Competing interests

The authors declare that they have no competing interests.

## Authors' contributions

RJ provides guidance and planning for this project. RJ and MW produced programs, analyzed main results and wrote the manuscript. MW and LL contributed in preparing some data and results analysis. All authors read and approved the final manuscript.

## Declarations

Publication of this article was funded by the National Basic Research Program of China (2012CB316504), the National High Technology Research and Development Program of China (2012AA020401), and the National Natural Science Foundation of China (61175002).

This article has been published as part of *BMC Genomics *Volume 16 Supplement 2, 2015: Selected articles from the Thirteenth Asia Pacific Bioinformatics Conference (APBC 2015): Genomics. The full contents of the supplement are available online at http://www.biomedcentral.com/bmcgenomics/supplements/16/S2

## References

[B1] Botstein D Risch N Discovering genotypes underlying human phenotypes: past successes for mendelian disease, future approaches for complex disease Nat Genet 2003 33 Suppl 228 237 12610532 10.1038/ng1090

[B2] Perez-Iratxeta C Bork P Andrade MA Association of genes to genetically inherited diseases using data mining Nat Genet 2002 31 3 316 319 12006977 10.1038/ng895

[B3] Meyre D Delplanque J Chevre JC Lecoeur C Lobbens S Gallina S Durand E Vatin V Degraeve F Proenca C Genome-wide association study for early-onset and morbid adult obesity identifies three new risk loci in European populations Nat Genet 2009 41 2 157 159 10.1038/ng.301 19151714

[B4] Bamshad MJ Ng SB Bigham AW Tabor HK Emond MJ Nickerson DA Shendure J Exome sequencing as a tool for Mendelian disease gene discovery Nature Reviews Genetics 2011 12 11 745 755 10.1038/nrg3031 21946919

[B5] Altshuler D Daly M Kruglyak L Guilt by association Nature genetics 2000 26 2 135 138 10.1038/79839 11017062

[B6] Emilsson V Thorleifsson G Zhang B Leonardson AS Zink F Zhu J Carlson S Helgason A Walters GB Gunnarsdottir S Genetics of gene expression and its effect on disease Nature 2008 452 7186 423 428 10.1038/nature06758 18344981

[B7] Tiffin N Kelso JF Powell AR Pan H Bajic VB Hide WA Integration of text- and data-mining using ontologies successfully selects disease gene candidates Nucleic acids research 2005 33 5 1544 1552 10.1093/nar/gki296 15767279 PMC1065256

[B8] Adie EA Adams RR Evans KL Porteous DJ Pickard BS Speeding disease gene discovery by sequence based candidate prioritization BMC bioinformatics 2005 6 55 10.1186/1471-2105-6-55 15766383 PMC1274252

[B9] Köhler S Bauer S Horn D Robinson PN Walking the interactome for prioritization of candidate disease genes The American Journal of Human Genetics 2008 82 4 949 958 10.1016/j.ajhg.2008.02.013 18371930 PMC2427257

[B10] Freudenberg J Propping P A similarity-based method for genome-wide prediction of disease-relevant human genes Bioinformatics 2002 18 Suppl 2 S110 115 10.1093/bioinformatics/18.suppl_2.S110 12385992

[B11] Turner FS Clutterbuck DR Semple CA POCUS: mining genomic sequence annotation to predict disease genes Genome biology 2003 4 11 R75 10.1186/gb-2003-4-11-r75 14611661 PMC329128

[B12] Lopez-Bigas N Ouzounis CA Genome-wide identification of genes likely to be involved in human genetic disease Nucleic acids research 2004 32 10 3108 3114 10.1093/nar/gkh605 15181176 PMC434425

[B13] Aerts S Lambrechts D Maity S Van Loo P Coessens B De Smet F Tranchevent LC De Moor B Marynen P Hassan B Gene prioritization through genomic data fusion Nat Biotechnol 2006 24 5 537 544 10.1038/nbt1203 16680138

[B14] Hamosh A Scott AF Amberger JS Bocchini CA McKusick VA Online Mendelian Inheritance in Man (OMIM), a knowledgebase of human genes and genetic disorders Nucleic acids research 2005 33 suppl 1 D514 D517 15608251 10.1093/nar/gki033 PMC539987

[B15] Lage K Karlberg EO Storling ZM Olason PI Pedersen AG Rigina O Hinsby AM Tumer Z Pociot F Tommerup N A human phenome-interactome network of protein complexes implicated in genetic disorders Nat Biotechnol 2007 25 3 309 316 10.1038/nbt1295 17344885

[B16] Wu X Jiang R Zhang MQ Li S Network-based global inference of human disease genes Mol Syst Biol 2008 4 189 18463613 10.1038/msb.2008.27 PMC2424293

[B17] Wu X Liu Q Jiang R Align human interactome with phenome to identify causative genes and networks underlying disease families Bioinformatics 2009 25 1 98 104 10.1093/bioinformatics/btn593 19010805

[B18] Li Y Patra JC Genome-wide inferring gene-phenotype relationship by walking on the heterogeneous network Bioinformatics 2010 26 9 1219 1224 10.1093/bioinformatics/btq108 20215462

[B19] Vanunu O Magger O Ruppin E Shlomi T Sharan R Associating genes and protein complexes with disease via network propagation PLoS computational biology 2010 6 1 e1000641 10.1371/journal.pcbi.1000641 20090828 PMC2797085

[B20] Chen Y Jiang T Jiang R Uncover disease genes by maximizing information flow in the phenome-interactome network Bioinformatics 2011 27 13 i167 176 10.1093/bioinformatics/btr213 21685067 PMC3117332

[B21] van Driel MA Bruggeman J Vriend G Brunner HG Leunissen JA A text-mining analysis of the human phenome European journal of human genetics: EJHG 2006 14 5 535 542 10.1038/sj.ejhg.5201585 16493445

[B22] Keshava Prasad TS Goel R Kandasamy K Keerthikumar S Kumar S Mathivanan S Telikicherla D Raju R Shafreen B Venugopal A Human Protein Reference Database--2009 update Nucleic acids research 2009 37 Database D767 772 18988627 10.1093/nar/gkn892 PMC2686490

[B23] Lindberg DA Humphreys BL McCray AT The Unified Medical Language System Methods of information in medicine 1993 32 4 281 291 8412823 10.1055/s-0038-1634945 PMC6693515

[B24] Allen AS Berkovic SF Cossette P Delanty N Dlugos D Eichler EE Epstein MP Glauser T Goldstein DB Han Y De novo mutations in epileptic encephalopathies Nature 2013 501 7466 217 221 10.1038/nature12439 23934111 PMC3773011

[B25] Aronson AR Effective mapping of biomedical text to the UMLS Metathesaurus: the MetaMap program Proceedings/AMIA Annual Symposium AMIA Symposium 2001 17 21 11825149 PMC2243666

[B26] Su AI Wiltshire T Batalov S Lapp H Ching KA Block D Zhang J Soden R Hayakawa M Kreiman G A gene atlas of the mouse and human protein-encoding transcriptomes Proc Natl Acad Sci USA 2004 101 16 6062 6067 10.1073/pnas.0400782101 15075390 PMC395923

[B27] Ashburner M Ball CA Blake JA Botstein D Butler H Cherry JM Davis AP Dolinski K Dwight SS Eppig JT Gene ontology: tool for the unification of biology. The Gene Ontology Consortium Nat Genet 2000 25 1 25 29 10.1038/75556 10802651 PMC3037419

[B28] Jiang R Gan M He P Constructing a gene semantic similarity network for the inference of disease genes BMC systems biology 2011 5 Suppl 2 S2 10.1186/1752-0509-5-S2-S2 22784573 PMC3287482

[B29] Gan M Correlating information contents of gene ontology terms to infer semantic similarity of gene products Computational and mathematical methods in medicine 2014 2014 891842 24963342 10.1155/2014/891842 PMC4054916

[B30] Kanehisa M Goto S KEGG: kyoto encyclopedia of genes and genomes Nucleic acids research 2000 28 1 27 30 10.1093/nar/28.1.27 10592173 PMC102409

[B31] UniProt C The Universal Protein Resource (UniProt) in 2010 Nucleic acids research 2010 38 Database D142 148 19843607 10.1093/nar/gkp846 PMC2808944

[B32] Li W McWilliam H Goujon M Cowley A Lopez R Pearson WR PSI-Search: iterative HOE-reduced profile SSEARCH searching Bioinformatics 2012 28 12 1650 1651 10.1093/bioinformatics/bts240 22539666 PMC3371869

[B33] Finn RD Mistry J Tate J Coggill P Heger A Pollington JE Gavin OL Gunasekaran P Ceric G Forslund K The Pfam protein families database Nucleic acids research 2010 38 Database D211 222 19920124 10.1093/nar/gkp985 PMC2808889

[B34] Snel B Lehmann G Bork P Huynen MA STRING: a web-server to retrieve and display the repeatedly occurring neighbourhood of a gene Nucleic acids research 2000 28 18 3442 3444 10.1093/nar/28.18.3442 10982861 PMC110752

[B35] Matys V Fricke E Geffers R Gossling E Haubrock M Hehl R Hornischer K Karas D Kel AE Kel-Margoulis OV TRANSFAC: transcriptional regulation, from patterns to profiles Nucleic acids research 2003 31 1 374 378 10.1093/nar/gkg108 12520026 PMC165555

[B36] Yang JJ Distribution of Fisher's combination statistic when the tests are dependent Journal of Statistical Computation and Simulation 2010 80 1 1 12 10.1080/00949650802412607

[B37] Storey JD The positive false discovery rate: A Bayesian interpretation and the q-value Annals of Statistics 2003 2013 2035

[B38] Benjamini Y Hochberg Y Controlling the false discovery rate: a practical and powerful approach to multiple testing Journal of the Royal Statistical Society Series B (Methodological) 1995 289 300

[B39] Haider S Ballester B Smedley D Zhang J Rice P Kasprzyk A BioMart Central Portal--unified access to biological data Nucleic acids research 2009 37 suppl 2 W23 W27 19420058 10.1093/nar/gkp265 PMC2703988

